# Structure and in vitro digestibility on complex of corn starch with soy isoflavone

**DOI:** 10.1002/fsn3.1896

**Published:** 2020-09-25

**Authors:** Siqi Wang, Tianhao Wu, Weijian Cui, Meihong Liu, Yuzhu Wu, Chengbin Zhao, Mingzhu Zheng, Xiuying Xu, Jingsheng Liu

**Affiliations:** ^1^ College of Food Science and Engineering Jilin Agricultural University Changchun China; ^2^ National Engineering Laboratory for Wheat and Corn Deep Processing Changchun China

**Keywords:** corn starch, glycemic index, *in vitro* digestibility, soy isoflavone, structural characterization

## Abstract

Rapid starch digestion rate is negative for the normal level of human blood glucose. This study investigated the protective effects of corn starch (CS) complexed with soy isoflavone (SI) on the control of starch digestibility and glycemic index (GI). The structure of the corn starch–soy isoflavone (CS‐SI) complexes was characterized by Fourier transform infrared (FT‐IR), X‐ray diffraction (XRD), Thermogravimetric analysis (TGA), and Differential scanning calorimetry (DSC), and the complexes digestibility was evaluated using in vitro digestion model. The results of FT‐IR spectrum showed that, compared with corn starch, new characteristic peaks were not occurred in CS‐SI complexes, and the value of R_1047/1022_ was decreased, which indicated the short‐range structure of CS‐SI complexes had been reduced. The V‐shaped structure characteristic peaks occurred obviously in CS‐SI complexes detected by XRD patterns, which formed a new crystalline structure. The thermal stability was improved in CS‐SI complexes revealed by TGA and DTG curves that the thermal cracking temperature increased from 315°C to 320°C. The enthalpy (*ΔH*) of CS‐SI complexes decreased from 2.34 J/g to 1.75 J/g showed by DSC data, which indicated that the ordered structure of starch was destroyed. Furthermore, the content of resistant starch increased from 10.53% to 21.78% and predicted glycemic index (pGI) reduced in CS‐SI complexes. In conclusion, the digestibility and pGI of starch can be improved by complexed with soy isoflavone.

## Graphical abstract

Soy isoflavone entered the spiral cavity of amylose through hydrophobic force, and a V‐shaped crystalline structure was formed in CS‐SI complexes, which increased SDS and RS content, and the starch digestibility and predicted glycemic index were reduced.

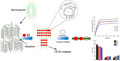

## 1 INTRODUCTION

Corn starch, one of the important components of corn, accounts for about 70% of the total mass usually. Starch has a positive effect on maintaining human life and movement, which play a key role obtaining energy from the diet for human. However, long‐term consumption of high glycemic index food was positively associated with obesity, hyperglycemia, diabetes typeⅡ, and other metabolic complications (Brand‐Miller et al., 2007), prompted researches to reduce digestibility and glycemic index of starch. The protein and phenolic compounds are regarded as safe, eco‐friendly, and effective substance that modified digestion and glycemic index of starch, effectively, by complexation of starch (Chi et al., 2018; Koh et al., 2010).

Flavonoids are considered as secondary metabolites of plants, with complex structure and high biological activity (Chen et al., 2014; Hoensch & Oertel, 2015; Ross & Kasum, 2002). Phenolic acids and flavonoids are the most abundant occupying approximately 30% and 60% of total phenolic compounds, respectively (Baiano & Del Nobile, 2016). Phenolic compounds could inhibit α‐amylase and glucosidase to weaken starch hydrolysis (Bordenave et al., 2014; Miao et al., 2015; Zhu, 2015), or forming a V‐shaped structure via hydrophobic interaction into the amylose helix cavity or hydrogen bonding to form a complex, starch digestibility, and pGI had been reduced (Cohen et al., 2011; Li et al., 2018).

Recent studies have shown that digestibility of starch could be controlled by complexed with phenolic compounds (Chi et al., 2017; Han et al., 2020). Quercetin interacted with corn starch, and the complexes formation was confirmed by the presence of the carbonyl signal around 1662/cm in the FT‐IR spectra. X‐ray diffraction revealed the crystal structure of native starch was disappeared and new crystalline regions were formed, and a new type of resistant starch was produced (Zhang et al., 2011). FT‐IR spectroscopy and XRD elucidated that lotus leaf flavonoid probably interacted with starch by non‐covalent bond especially hydrogen bond, and lotus leaf flavonoid–starch complexes exhibited inhibitory effect on starch digestion (Wang et al., 2018). Graft of quercetin and starch was proved by Fourier transform infrared spectroscopy, and proton signal of nuclear magnetic resonance and RS content significantly increased (Liu et al., 2018). However, few reports mentioned starch digestibility and glycemic index were regulated by flavonoid compounds such as soy isoflavone.

In this study, the structure of CS‐SI complexes was characterized by analysis of FT‐IR, XRD, TGA, and DSC, and the digestibility of complexes was evaluated using an in vitro digestion model. This research may provide new information on the properties of starch–flavonoid complexes and help to develop starch‐based foods containing flavonoid compounds.

## 2 MATERIALS AND METHODS

### 2.1 Materials

Corn starch (83.42% total starch, 28.30% amylose, 0.54% fat, 0.36% protein, and 11.35% moisture) was purchased from Shanghai Jinsui Biological Technology Co., LTD company. Soy isoflavone (SI, ≥90%) was purchased from Xian Tianfeng Biotechnology Co., LTD company. α‐amylase from porcine pancreas (8 × USP) and glucosidase from aspergillus niger (≥260 U/ml) were purchased from Sigma‐Aldrich. D‐glucose assay kit was purchased from Megazyme International Ireland. Other reagents were of analytical grade.

### 2.2 Preparation of CS‐SI complexes

The preparation of CS‐SI was slightly modified with reference to the published method (Li, et al., 2018). 10% starch dispersion was configured (w/v), 0%, 0.5%, 1%, 1.5%, and 2% of SI (SI/CS, w/w) dissolved in ethanol, and added dropwise to the starch dispersion within 30 min with constant stirring. After incubating at 70°C for 1 hr, the mixture was slowly cooled. The resulting suspension was centrifuged at 1734 *g* for 5 min, washed three times with ethanol solution (50%, v/v), and centrifuged to remove soy isoflavone that were not complexed with corn starch, and the samples were freeze‐dried. The samples obtained are marked as SSI‐0, SSI‐1, SSI‐2, SSI‐3, and SSI‐4.

### 2.3 Determination of SI content in the complexes

Refer to published methods for minor modifications (Li, et al., 2018). The sample (20 mg, dry basis) was dissolved in 3 ml of dimethyl sulfoxide (DMSO), centrifuged for supernatant, 2 ml of solution with 200 μl of 95% ethanol, and diluted to volume with deionized water. The SI content was determined by measuring absorbance at 260 nm.

### 2.4 Fourier transform infrared spectroscopy

The samples structure was determined by using Fourier infrared spectrometer, and published methods for minor modifications were referred (Zhao et al., 2020). Mixed with dried potassium bromide (KBr), samples were ground under the infrared lamp. After subtracting the background of KBr, the FT‐IR spectra ranging from 400 to 4,000/cm were recorded with 4/cm resolution. Before FT‐IR measurement, the samples were equilibrated at 40°C for 24 hr.

### 2.5 X‐ray diffractometry

Method of X‐ray diffraction referring to published research for minor modifications was applied to determine samples crystal structure (Lopez‐Rubio et al., 2008). The samples were laid flat on the groove and compressed with a tablet, running at 40 kV and 40 mA, and were scanned in the range from 4 to 40°(2θ), scanning speed 5°/min.

### 2.6 Thermogravimetric analysis

Accurately weigh 5.0 mg of sample into the crucible, as a comparison with empty crucible. Thermogravimetric synchronous thermal differential analyzer increased the temperature from 25.0°C to 600.0°C at a heating rate of 10°C/min under a nitrogen atmosphere. TGA and differential thermogravimetry (DTG) curves of samples were recorded.

### 2.7 Differential scanning calorimetry

Using DSC to determine the thermal properties of samples, refer to published methods for minor modifications (He et al., 2018). Accurately weigh 3.0 mg sample was mixed with deionized water in the ratio of 1:3, sealed gland, and placed at 4°C environment to equilibrate for 24 hr, temperature measurement with differential scanning calorimeter and used empty crucible as a comparison. The temperature rising range has been set 30°C ～ 120°C, with the heating rate 10°C/min. From the DSC data, the initial temperature of gelatinization *T_0_*, the peak temperature of gelatinization *T_p_*, the final temperature of gelatinization *T_C_*, and the enthalpy value *ΔH* were known.

### 2.8 In vitro digestion

Based on the Englyst method with slight modification (Englyst, Kingman, & Cummings, 1992), 1 g of sample was suspended in sodium acetate buffer solution (20 ml, 0.1 M, pH5.2), after equilibrating at 37°C for 10 min, 5 ml of mixed enzyme solution (a‐amylase and glucosidase) and 5 glass balls were added to each conical flask. Then, the samples were placed at shaking bath (37°C, 190 rpm) for enzymatic hydrolysis. 0.5 ml sample was mixed with 20 ml of absolute ethanol (70%, v/v) for stopping the reaction at different times (0, 10, 20, 30, 40, 50, 60, 80, 100, and 120 min), centrifuged for 10 min at 4,000 *g*. The hydrolysate of centrifuged supernatant was measured by GOPOD reagent. The glucose content after 0, 20, and 120 min of hydrolysis was marked as G0, G20, and G120, respectively. The content of rapidly digested starch (RDS), slowly digestible starch (SDS), and resistant starch (RS) of CS‐SI complexes was calculated by the following formula:

$$\text{RDS}/\%=\frac{0.9\times \left(G20‐G0\right)}{\text{TS}}\times 100$$

$$\text{SDS}/\%=\frac{0.9\times \left(G120‐G20\right)}{\text{TS}}\times 100$$

RS/%=TS‐RDS‐SDS

In the formula, TS represents the total starch content used to measure digestibility. Herein, TS equals 1 g.

### 2.9 Hydrolysis kinetic and predicted glycemic index

A first order kinetic model [C = C_∞_ (1‐e^−kt^)] was applied to investigate hydrolysis kinetics and pGI of the CS‐SI complexes (Gorii’ et al., 1997), where C, C_∞_, and k represented the percentage of starch hydrolyzed, the maximum hydrolysis extent and the kinetic constant, respectively, at time *t* (0, 10, 20, 30, 40, 50, 60, 80, 100, and 120 min).The hydrolysis index (HI) is the ratio of the area under hydrolysis curve (AUC) of samples to the AUC of fresh white bread. According to the equation, pGI = 8.198 + 0.862HI, get pGI (Kitts & Wijewickreme, 1994).

### 2.10 Statistic analysis

All data are the average of 3 parallel measurements, and the results are expressed in x ± s. The related charts are drawn using Origin 2018 software (Origin Lab Corporation), and the variances are calculated using SPSS 21 software (SPSS Inc). Analysis measurement, the difference is statistically significant. The significant differences were evaluated and displayed by different letters.

## 3 RESULTS AND DISCUSSION

### 3.1 FT‐IR spectrum analysis of CS‐SI complexes

**Figure 1:**
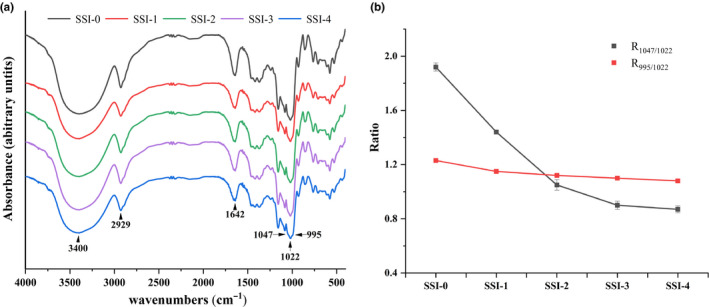
(a) FT‐IR spectra of corn starch–soy isoflavone complexes. (b) R_1047/1022_ and R_995/1022_ values of corn starch–soy isoflavone complexes

FT‐IR spectroscopy was performed to investigate the structure of CS‐SI complexes, as Figure 1a shown, and the ‐OH stretching vibration absorption peak is between 3,600/cm and 3,100/cm and the most intense appeared at 3,400/cm. The sharp band at 2,929/cm refers to the anti‐symmetrical stretching vibration of ‐CH_2_ present in starch molecule. The band at 1642/cm is attributed to the C = O stretching vibration in a carbohydrate group. Strong absorption peak appeared in all samples. Compared with SSI‐0, in the spectral range of 4,000 ~ 400/cm, the shape and position of infrared absorption peaks were similar and offset not obviously, new characteristic peaks not appeared or disappeared, which indicated that new group not generated in CS‐SI complexes and they were not combined by a covalent bond.

FT‐IR spectroscopy has been widely applied to investigate the short‐range molecular structure changes in starch double helices, and the differences in the crystalline and amorphous regions were observed between complexes. The ratio of FT‐IR spectrum (1045/1022)/cm and (995/1022)/cm absorption peak intensity can reflect the change of ordered structure in starch. The characteristic of ordered structure in aggregated structure of starch crystalline region is attributed to absorption peak near 1,054/cm, the characteristic of random coil structure of starch in the amorphous region is represented to absorption peak near 1,020/cm, and the absorption peak near 995/cm is an ordered structure of hydrogen bonds formed by the hydroxyl groups of anhydroglucose units between starch molecules (Shingel, 2002; van Soest et al., 1995). Phenolic compounds are believed to hinder the formation of starch order structure, thereby inhibiting retrogradation of starch (Wu et al., 2009). The values of R_1045/1022_ and R_995/1022_ decreased, shown in Figure 1b, indicated that the amorphous phase structure in starch and distance between starch molecular chains were increased, the formation of hydrogen bonds between starch molecules was reduced, and the production of a relatively ordered structure of starch was hindered by complexed soy isoflavone.

### 3.2 XRD analysis of CS‐SI complexes

**Figure 2:**
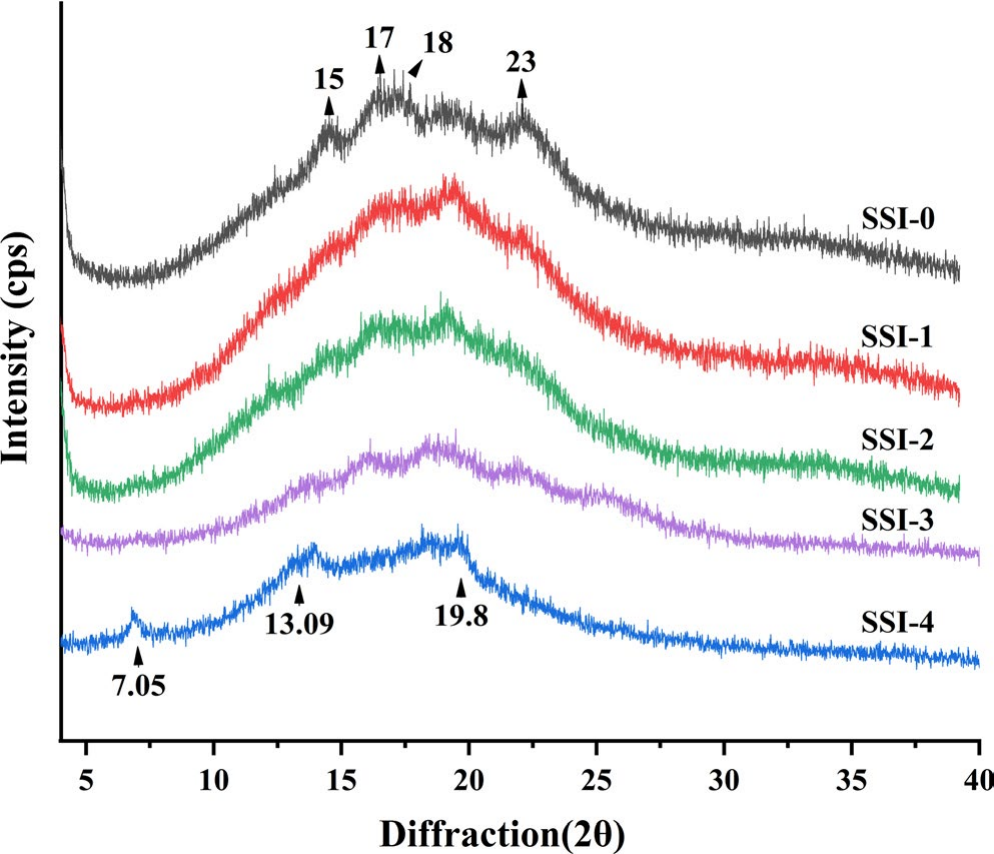
XRD patterns of corn starch–soy isoflavone complexes

XRD analysis was performed to investigate the crystal structure of CS‐SI complexes. Starch regards as a polycrystalline system with crystal structure A,B,C,V, but V‐type structure is rarely in natural starch. The strong diffraction peaks at 15°, 17°, 18,° and 23° appeared in A‐type crystals, and near 7.5°,12.8°, and 19.8°,V‐type crystals have strong diffraction peaks (Sun et al., 2015). As shown in Figure 2, corn starch exhibited intense peaks at 15°, 17°, 18°, and 23°, indicated that the crystal type of maize starch belonged to a classical A‐type. The samples had both A‐type and V‐type crystal structures, when a low concentration of SI was added. With increasing the addition of SI, the type A crystal structure was gradually disappeared. SSI‐4 had diffraction peaks at 7.05°, 13.09°, and 19.8°, which were typical of V_7_ helical‐type diffraction (Liu et al., 2019), and its crystal structure was transformed into a V‐shaped structure, completely. The soy isoflavone might enter the spiral cavity of amylose through hydrophobic force as a guest, a single spiral chain consisting of 7 glucose units formed in amylose molecules, soy isoflavone might be located inside spiral cavity or the hydroxyl group of soy isoflavone complexed with two helices, then participated in starch recrystallization. During the complexing process, the crystallization area of starch was destroyed, the original crystal structure of starch granules was lost, and a unique V‐shaped crystal structure was formed (Chang, He, & Huang, 2013). The results further indicated the formation of complexes. The crystallinity was decreased and the amorphous region increased, and starch molecular structure tended to be disordered. In addition, the relative peak intensity of V‐shaped crystal structure was affected by SI content. Related research showed that V‐shaped crystal structure was more conducive to the formation of resistant starch and played an important role in inhibiting starch digestion (Tan, 2020).

### 3.3 Thermogravimetric analysis of CS‐SI complexes

**Figure 3:**
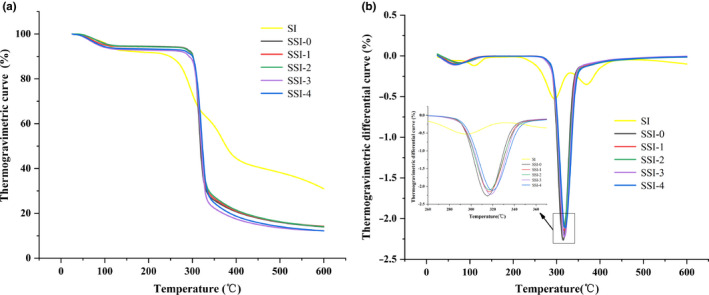
(a) Thermogravimetric curve of corn starch–soy isoflavone complexes. (b) Thermogravimetric differential curve of corn starch–soy isoflavone complexes

To further investigate the thermal stability of CS‐SI complexes, thermogravimetric analysis was performed. The thermogravimetric curves of SI and CS‐SI complexes were shown in Figure 3a. From TGA curve, it could be seen that SI had four stages of quality decline, and the complexes had three stages of quality decline. The weight loss of SI, in the first stage, was caused by loss of water molecules. In the second stage, the decomposition of SI molecules could lead to a significant decrease in quality. The weight loss of the third and fourth stages, might be due to decomposition of SI residues, and decomposed SI molecules need a period of time to reach a constant weight, so that the mass loss slowly (Zhang et al., 2011).CS‐SI complexes had slight weight loss in the temperature range of 40 ~ 150°C, indicated the disappearance of water. An obvious weightlessness step was formed at 260 ~ 360°C and then entered the stage of slow weightlessness, complexes decomposed and lost weight rapidly. At this time, the depolymerization reaction of starch macromolecules formed glucose and a series of lower molecular weight gaseous products; as temperature increased, the glycosylation of glucose and some polyhydroxy groups would also decompose. As compared with SSI‐0, CS‐SI complexes had lower weight loss at decomposition stage.

The thermogravimetric differential curves of SI and CS‐SI complexes were shown in Figure 3b, and the maximum peak in curve represented maximum decomposition rate of samples. SI had three weightlessness peaks, and CS‐SI complexes had two obvious weightlessness peaks, which represented their weightlessness stages. Compared with SSI‐0, decomposition temperature of CS‐SI complexes increased from 315°C to 320°C. The decomposition temperature of starch is related to its crystal type, processing method, chemical modification, and relative molecular mass, and high decomposition temperature proves stronger thermal stability (Liu & Wang, 2017). In this study, the thermal stability of starch was improved after adding soy isoflavone. In addition, in the decomposition stage, the weight loss rate of CS‐SI complexes was lower than SSI‐0.

### 3.4 Thermal properties analysis of the CS‐SI complexes

Starch gelation and phase change can be quantitatively analyzed by DSC. Table 1 was shown the thermal characteristic parameters of CS‐SI complexes, starting temperature *T_0_*, peak temperature *T_c_*,ending temperature *T_p_*, and enthalpy value *ΔH*. Compared with SSI‐0, *T_0_* increased significantly (*p* < .05); *T_p_* and *T_c_* both showed an upward trend. The increase in gelatinization temperature could be attributed to interaction between amylose–amylose or amylose–amylopectin chains, and the formation of complexes between amylose and other substances. In our present research, ‐OH groups in SI might bind to starch granules, the redistribution of water between soy isoflavone and corn starch, which in turn affected the starch gelatinization transformation and hydration of amorphous region in starch granules, resulted in starch gelatinization temperature increased.

**Table 1:** Thermal properties of corn starch–soy isoflavone complexes

Samples	*T_0_*(°C)	*T_p_*(°C)	*T_c_*(°C)	*ΔH* (J/g)
SSI−0	70.01 ± 0.02^a^	73.28 ± 0.04^a^	77.30 ± 0.18^a^	2.34 ± 0.07^e^
SSI−1	70.15 ± 0.08^b^	73.29 ± 0.02^a^	77.85 ± 0.04^b^	2.22 ± 0.04^d^
SSI−2	70.3 ± 0.01^c^	73.77 ± 0.06^b^	77.85 ± 0.03^b^	1.85 ± 0.01^c^
SSI−3	71.46 ± 0.02^d^	73.78 ± 0.06^b^	77.41 ± 0.01^a^	1.78 ± 0.04^b^
SSI−4	72.48 ± 0.04^e^	73.80 ± 0.08^b^	77.47 ± 0.04^a^	1.75 ± 0.02^a^

Note

Values within column with different superscript letters are significantly different (*p* < .05). SSI‐0, SSI‐1, SSI‐2, SSI‐3, and SSI‐4 represent corn starch with 0%, 0.5%, 1.0%, 1.5%, and 2.0% soy isoflavone (w/w), respectively.

The enthalpy value (*ΔH*) reflects the energy required for destruction of starch double helix during starch gelatinization, and before gelatinization of starch, measures the order of molecular chains inside starch granules. The destruction of ordered structure in starch granules will lead to a decrease in enthalpy value. The data in Table 1 were shown that as the concentration of soy isoflavone increased, *ΔH* of CS‐SI complexes decreased significantly (*p* < .05). Similar to the result of Han (Han & Koh, 2011). This might be due to addition of soy isoflavone which formation of ordered starch structures was disrupted, resulted in *ΔH* decreased. This phenomenon supported the results of FT‐IR. SI contained ‐OH groups would interact with starch, which could disturb hydrogen bonds formed between starch chains, thus starch crystallization reduced. CS‐SI complexes crystalline area reduced might also cause this phenomenon (Han & Koh, 2011; Zheng et al., 2020).

### 3.5 In vitro digestibility of the CS‐SI complexes

**Figure 4:**
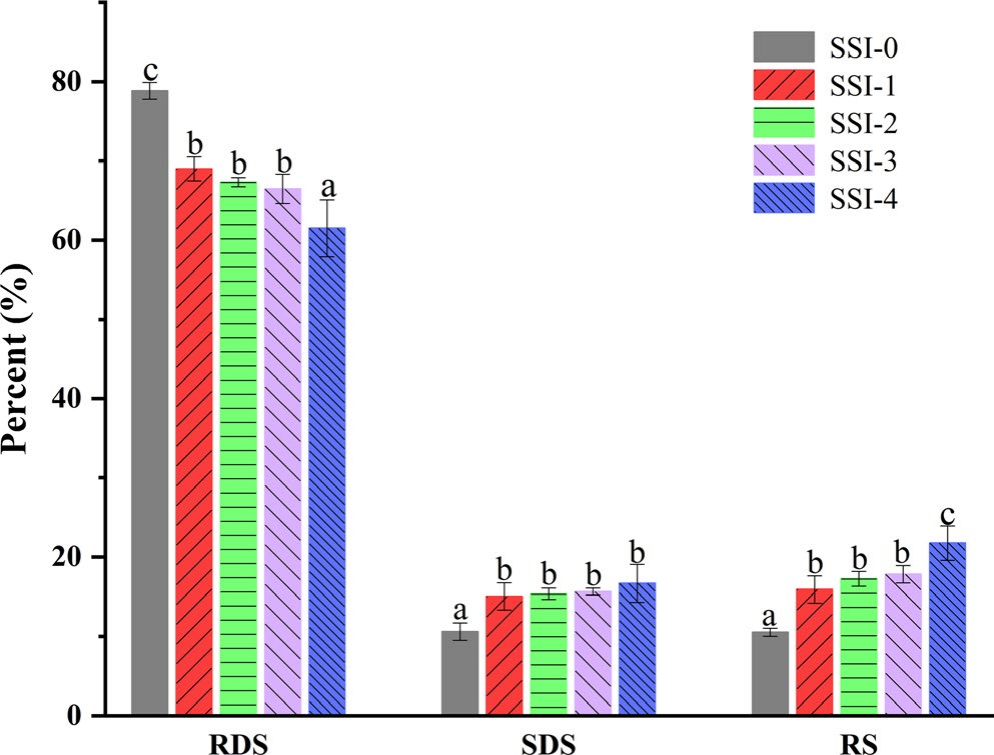
RDS, SDS and RS contents of corn starch–soy isoflavone complexes

Resistant starch is considered to be a dietary fiber that helps lower blood sugar, and a higher RS content and lower GI may be effective in preventing metabolic syndromes such as obesity and diabetes. The interactions between starch and phenolic compounds can affect in vitro digestion of starch and its postprandial blood glucose response. It had been proposed that phenolic compounds reduced starch digestibility in two ways: inhibit the activity of enzymes or change the structural characteristics of starch to weaken the effect of amylase (Chi et al., 2017). Many factors also influenced starch digestibility: crystal type, ratio of amylose and amylopectin, particle size, etc (Gularte et al., 2012; Tian et al., 2019). According to different degrees of digestibility, starch is classified as fast digestion starch, slow digestion starch, and resistant starch. The content of RDS, SDS, and RS in CS‐SI complexes is shown in Figure 4. The CS‐SI complexes showed inhibitory effect against starch digestion and potential health benefits to humans, compared to starch without SI. SSI‐0 had the highest RDS content (78.86 ± 1.05%), and RDS content of CS‐SI complexes gradually decreased, followed by SSI‐1 (69.01 ± 1.51%) >SSI‐2 (67.34 ± 0.57%) >SSI‐3 (66.49 ± 1.85%) >SSI‐4 (61.51 ± 3.61%). The SDS content of SSI‐0 was 10.61 ± 1.10%, and RS content was 10.53 ± 0.51%. Compared with SSI‐0, the SDS and RS of complexes gradually increased, and the SDS content increased from 15.05 ± 1.72% to 16.70 ± 2.40%, the RS content increased from 15.93 ± 1.72% to 21.78 ± 2.18%. As RDS, SDS, and RS content shown, the digestibility of starch was improved and content of starch SDS and RS was increased with soy isoflavone. This might be because soy isoflavone entered starch spiral inside when complexed with starch, the crystal structure of starch was changed, starch structure was more reliable, accessibility of hydrolytic enzymes to starch chains was restricted, thus enzymatic hydrolysis of amylase was hindered, therefore starch digestion was inhibited.

### 3.6 Predicted glycemic index of CS‐SI complexes

**Figure 5:**
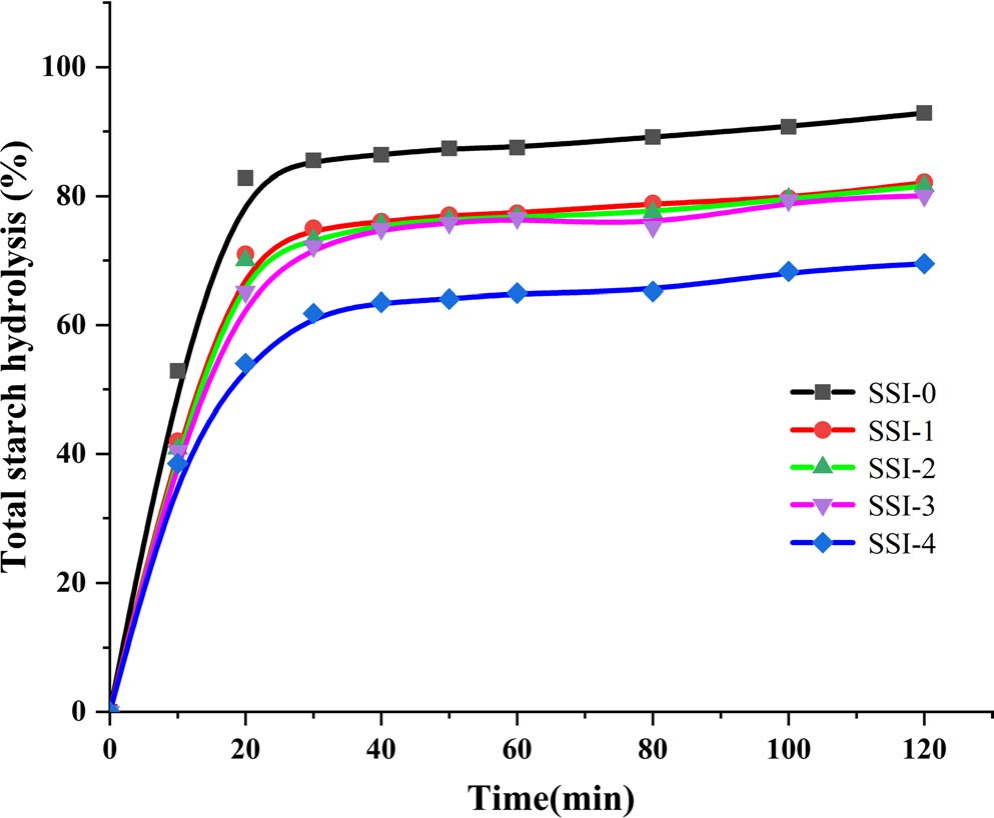
In vitro digestion of corn starch–soy isoflavone complexes

The typical digestion curves of CS‐SI complexes in Figure 5, the hydrolysis kinetic parameters and pGI of CS‐SI complexes in Table 2. Compared with SSI‐0, hydrolysis rate (HI) value of CS‐SI complexes decreased gradually, and the difference was significant (*p* < .05), indicated that digestibility of corn starch after complexing with soy isoflavone was significantly reduced. The maximum hydrolysis degree C_∞_ of CS‐SI complexes ranged from 69.482 to 82.098, which was significantly lower than SSI‐0 (92.875). It is further shown that starch hydrolysis rate was reduced complexed with soy isoflavone, thereby digestibility of starch was reduced. In addition, the hydrolysis kinetic constant *K* value was decreased gradually. V‐shaped crystal structure of starch shows more resistance to α‐amylase hydrolysis, and the rate of starch hydrolysis can be reduced (Jane & Robyt, 1984).

**Table 2:** Digestibility, model parameters, calculated hydrolysis indices, and predicted glycemic indices (pGI) of corn starch–soy isoflavone complexes

Samples	C_∞_	k	HI	pGI
SSI−0	92.875^c^	0.04^a^	97.87^c^	92.56^c^
SSI−1	82.098^b^	0.037^a^	85.62^b^	82.00^b^
SSI−2	81.576^b^	0.036^a^	84.68^b^	81.19^b^
SSI−3	80.060^b^	0.035^a^	83.27^b^	79.98^b^
SSI−4	69.482^a^	0.034^a^	71.57^a^	68.09^a^

Note

(Different letters indicated significant differences in each column at 0.05 level). SSI‐0, SSI‐1, SSI‐2, SSI‐3, and SSI‐4 represent corn starch with 0%, 0.5%, 1.0%, 1.5%, and 2.0% soy isoflavone (w/w), respectively.

SSI‐0 had the highest pGI value (92.56), as the concentration of soy isoflavone increased, pGI of CS‐SI complexes decreased gradually, and SSI‐4 had the lowest pGI value (68.09). Relatively higher SDS and RS content and lower pGI values in SSI‐1, SSI‐2, and SSI‐3, however they were still considered high GI (GI > 75) foods, while SSI‐4 was considered as medium GI food (Babatola et al., 2020). Similar to Chi results (Chi et al., 2019), SSI‐4 had the lowest digestibility and the highest RS, and the largest reduction of HI and pGI was achieved for SSI‐4. Change in HI, digestibility, and pGI was due to crystal structure and inhibition of amylase activity in CS‐SI complexes (Chi et al., 2019), indicated that pGI of starch reduced by soy isoflavone and showed a concentration‐dependent. Therefore, it can be used as a new approach for regulating starch digestibility and glucose production complexed with soy isoflavone. In addition, the analysis of dose–response relationship is necessary to quantitatively manipulate postprandial glycemic response.

## 4 CONCLUSION

In this experiment, structural and in vitro digestion characteristics of corn starch incorporated with soy isoflavone were analyzed systematically, and glycemic index was predicted. The change mechanism of digestibility in CS‐SI complexes was revealed, and the non‐covalent interaction between corn starch and soy isoflavone was verified. CS‐SI complexes lost its original crystal, and a unique V‐shaped crystal structure was formed. By complexed with soy isoflavone, short‐range ordered structure and enthalpy value of starch were reduced, thermal stability of starch was improved, the content of SDS and RS in starch was increased, and pGI was decreased. From the structure characterization and digestibility results of CS‐SI complexes, it could be concluded that soy isoflavone entered amylose spiral cavity through hydrophobic force, and digestibility of starch was reduced by the change of CS‐SI complexes crystal structure. Therefore, complexed with soy isoflavone will open a pathway to modulate digestibility of starch.

## CONFLICT OF INTEREST

The authors declare no conflict of interest.

## Data Availability

Author elects to not share data. Research data are not shared.
